# Remimazolam-based anesthesia with intraoperative motor evoked potential monitoring in a patient with Leigh syndrome undergoing scoliosis surgery: a case report

**DOI:** 10.1186/s40981-026-00851-x

**Published:** 2026-02-13

**Authors:** Takahiro Kuwabara, Takahiro Tamura, Masashi Takakura, Tasuku Fujii, Kanako Ozeki, Koichi Akiyama

**Affiliations:** 1 Department of Anesthesiology, Nagoya University Hospital, Nagoya, Japan; 2 Department of Anesthesiology, Nagoya University Graduate School of Medicine, 65 Tsurumai-cho, Showa-Ku, Nagoya, 466-8550 Japan

**Keywords:** Leigh syndrome, Remimazolam, Motor evoked potential monitoring, Mitochondrial encephalomyopathy

## Abstract

**Background:**

We present a case of scoliosis surgery performed under general anesthesia with remimazolam in a 16-year-old patient with Leigh syndrome (LS), a subtype of mitochondrial encephalomyopathy. Anesthetic management in such patients is challenging because of the risks of malignant hyperthermia with inhalational agents and propofol infusion syndrome, and because many of these patients present with impaired consciousness and respiratory compromise, anesthetic management becomes extremely difficult. To date, very few reports have described remimazolam use in LS, and none have described cases requiring intraoperative motor-evoked potential (MEP) monitoring.

**Case presentation:**

A patient diagnosed with LS at 7 months of age underwent corrective scoliosis surgery. Anesthesia was induced and maintained using remimazolam supplemented with opioids and muscle relaxants. Some intraoperative MEP signals were attenuated but remained monitorable. The patient was extubated with flumazenil and admitted to the intensive care unit, where a transient decrease in oxygenation was observed. However, the patient recovered without any complications and was discharged uneventfully.

**Conclusion:**

Remimazolam may be a feasible anesthetic option for patients with LS undergoing surgery requiring MEP monitoring. However, its use should be carefully determined based on factors such as the patient’s age, level of consciousness, respiratory function, and history of epilepsy.

## Background

Leigh syndrome (LS) is a subtype of mitochondrial encephalomyopathy [1, 2], typically presenting in infancy with seizures, status epilepticus, developmental delay, dysarthria, and ataxia [3, 4]. Affected patients often experience recurrent episodes of lactic acidosis that may progress to respiratory failure and death, with approximately 50% dying before 3 years of age [3, 5, 6]. LS is a rare disorder with an estimated prevalence of approximately one in 40,000 individuals. Reports on general anesthesia in these patients are limited, and anesthetic management remains particularly challenging due to the risk of malignant hyperthermia with inhaled anesthetics and propofol infusion syndrome (PRIS).

Moreover, volatile anesthetics markedly suppress intraoperative motor-evoked potentials (MEPs), which are crucial for neurological monitoring during scoliosis surgery. This further limits anesthetic options for patients requiring spinal surgery with neurophysiological monitoring. We present a case of scoliosis surgery in a patient with LS, in whom general anesthesia was successfully managed with remimazolam. Although remimazolam use has been reported in pediatric and adult patients with mitochondrial diseases [7–9], most cases involve mitochondrial myopathy, encephalopathy, lactic acidosis, and stroke-like episodes. To the best of our knowledge, reports on the use of remimazolam for general anesthesia in patients with LS are extremely limited, and none have described its use during MEP monitoring.

## Case presentation

**Fig. 1:**
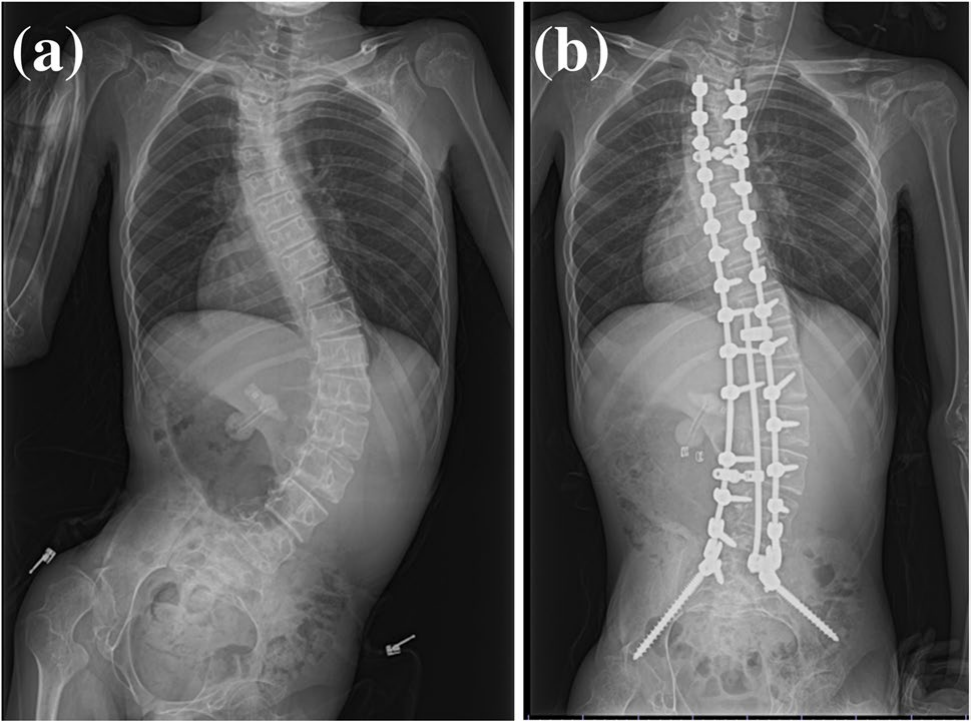
Preoperative and postoperative chest and abdominal radiographs images. (**a**) Preoperative radiograph showing severe neuromuscular scoliosis with marked thoracic deformity and reduced thoracic volume. (**b**) Postoperative radiograph demonstrating correction of spinal alignment after posterior spinal fusion from T3 to the pelvis. The patient presented with severe three-dimensional spinal deformity, with a Cobb angle of 76° and pelvic tilt of 38°. Compression of the lungs was also observed due to thoracic deformity associated with scoliosis

A 16-year-old boy (height, 148 cm; weight, 24 kg) was born at 37 weeks of gestation with a birth weight of 2102 g and experienced neonatal asphyxia. At 7 months of age, he was noted to have poor head control and an inability to roll over, and further evaluation led to a diagnosis of LS. At approximately 10 years of age, the patient developed influenza encephalopathy and became bedridden. Progressive neuromuscular scoliosis necessitated a posterior corrective fusion surgery extending from T3 to the pelvis. His regular medications included levocarnitine, ubidecarenone, triclofos sodium, clonazepam, perampanel hydrate, levetiracetam, and various vitamin supplements. The results of routine preoperative studies were unremarkable. The patient was classified as having an ASA (American Society of Anesthesiologists) physical status of III. Preoperatively, the level of consciousness fluctuated; during awake periods, the patient was able to communicate “yes” or “no” through finger movements, but meaningful communication was generally difficult. Regarding motor function, a marked limitation of range of motion was observed in all extremities, predominantly in the left elbow and right knee. The left elbow could not be extended, and the left shoulder could not be elevated. Manual muscle testing (MMT) could not be performed because of contractures and difficulty in communication; however, there was no paralysis, and spontaneous movements were observed within a limited range of motion. The patient had no family history of malignant hyperthermia. Preoperative and postoperative chest and abdominal radiographs images are shown in Fig. 1.

**Fig. 2:**
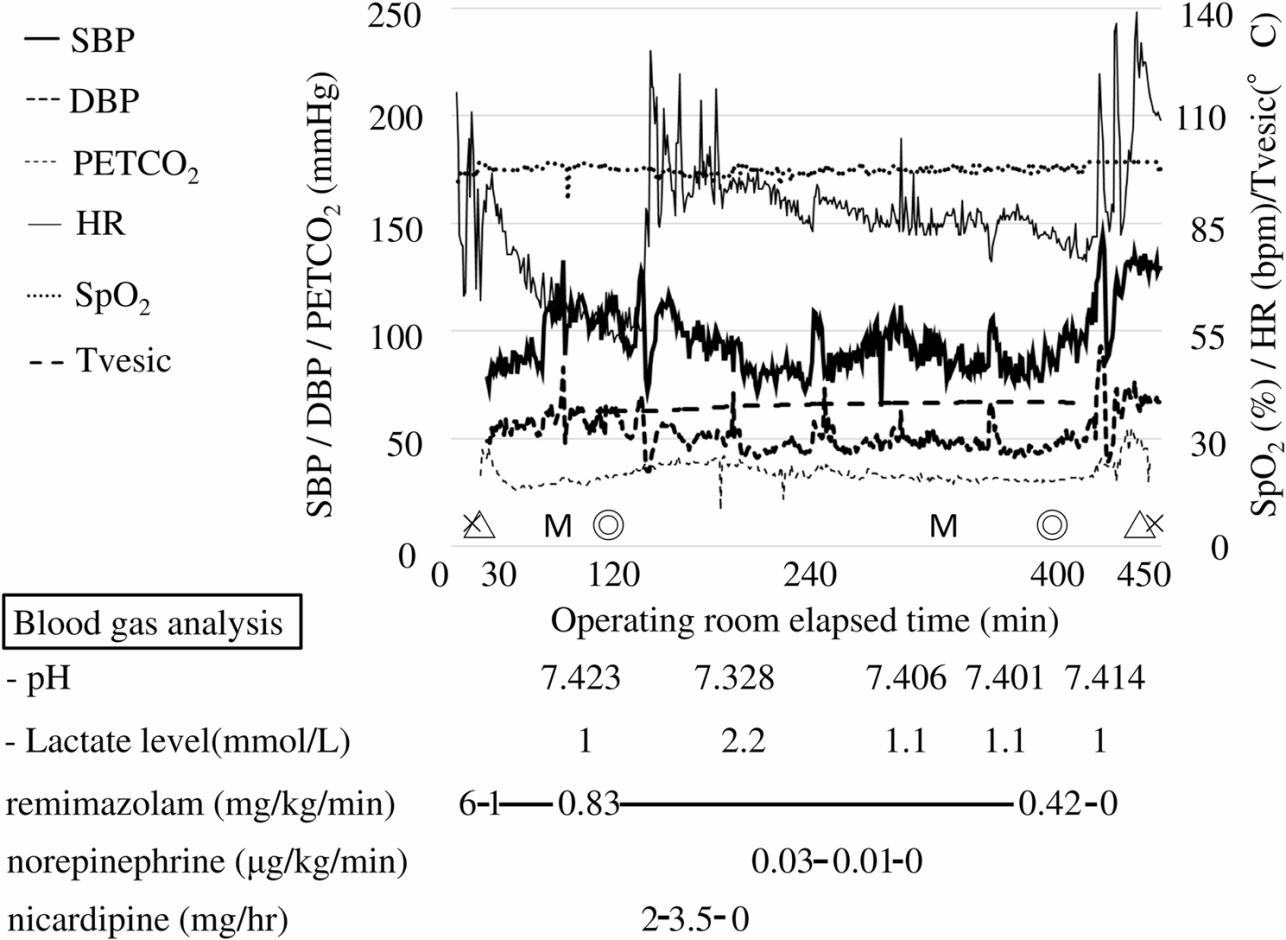
The anesthesia chart. Intraoperative anesthetic and hemodynamic course during scoliosis surgery under remimazolam anesthesia with MEP monitoring. Changes in systolic blood pressure (SBP), diastolic blood pressure (DBP), heart rate (HR), oxygen saturation (SpO₂), end-tidal carbon dioxide (PETCO₂), and vesical temperature (T_vesic) over time. Beats per minute were abbreviated as bpm. X indicates the start and end of anesthesia; △ indicates tracheal intubation and extubation; ◎ indicates the start and end of surgery. M denotes the timing of the representative MEP recordings, corresponding to the initial baseline measurement after induction and the final measurement before the completion of surgery. Eight measurements were performed, including the initial baseline and final measurements

The anesthetic course is shown in Fig. 2. General anesthesia was induced with remimazolam at 6 mg/kg/hr. After confirming the loss of spontaneous movement, fentanyl 15 µg and rocuronium 15 mg were administered, followed by tracheal intubation using a 6.0-mm tube and a McGrath videolaryngoscope. Remimazolam infusion was reduced to 1 mg/kg/hr. Intraoperatively, anesthesia was maintained with remimazolam at 0.83 mg/kg/hr and remifentanil at 0.35–0.42 µg/kg/min, while monitoring the SedLine (Masimo Corporation; Irvine, CA, USA) values.

**Fig. 3:**
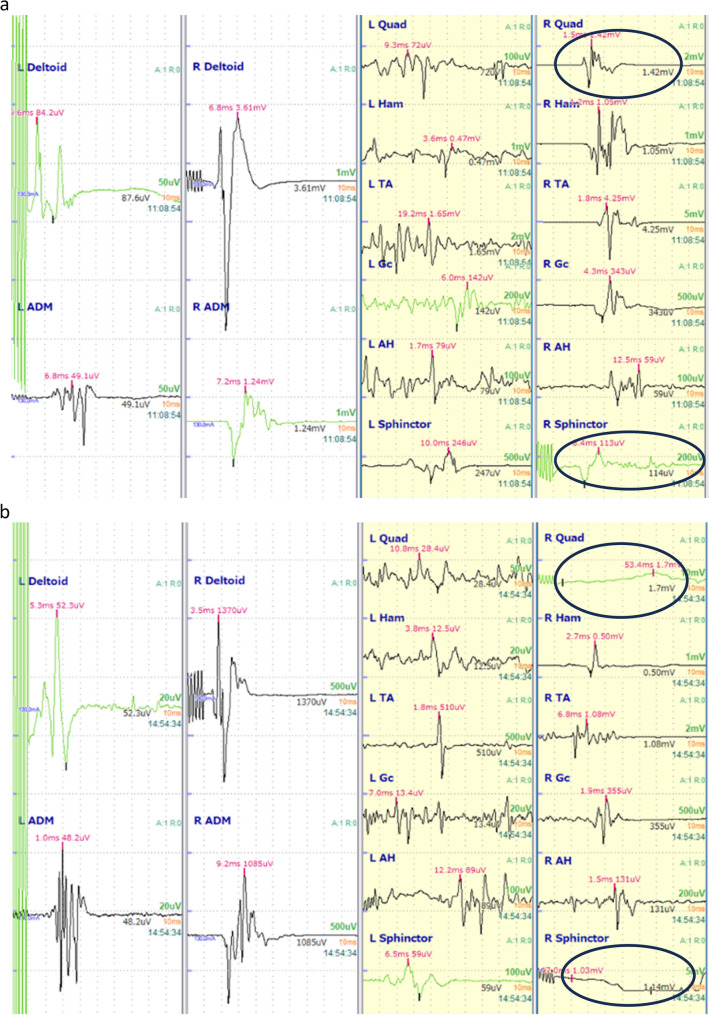
Representative transcranial motor evoked potential waveforms recorded during surgery. (**a**) The panel shows baseline transcranial motor- evoked potential (Tc-MEP) waveforms obtained after induction of anesthesia and before surgical manipulation. (**b**)The panel displays Tc-MEP waveforms recorded at the end of surgery, following the completion of surgical maneuvers associated with a risk of spinal cord injury. MEP signals from the right quadriceps femoris and anal sphincter muscles became partially unobtainable (indicated by circles); however, overall waveform monitoring remained feasible. The amplitudes of each waveform are summarized in Table 1

In this case, the patient presented with a severe three-dimensional spinal deformity with a Cobb angle of 76° and a pelvic tilt of 38°. Owing to the extensive nature of the surgery, the risk of intraoperative spinal cord injury is high; therefore, MEP monitoring was continuously performed throughout the procedure. Transcranial stimulationwas applied from C3 to C4 using a bipolar electrical stimulator (Neuromaster MEE-2000; Nihon Kohden, Tokyo, Japan). Five trains of stimuli were delivered, each consisting of five consecutive pulses. The pulse width was 0.5 ms, the interstimulus interval was 2.0 ms, and the stimulation intensity was 130 mA. Recording electrodes were placed in the deltoid, abductor digiti minimi, quadriceps femoris, hamstring, tibialis anterior, gastrocnemius, abductor hallucis brevis, and anal sphincter muscles. The initial and final intraoperative MEP waveforms are shown in Fig. 3, and the corresponding amplitudes are summarized in Table 1. Although MEP recordings from the right quadriceps and sphincter were lost approximately one hour after the start of surgery, overall MEP monitoring remained feasible and stable, with some lower-limb muscles showing amplitude reductions > 70%.

**Table 1:** Comparison of MEP waveforms between the initial and the final intraoperative recordings

	Deltoid	ADM	Quad	Ham	TA	Gc	AH	Sphincter
Left								
Initial waveform	88	49	72	466	1648	142	79	246
Postoperative waveform	52	48	28	13	510	13	89	59
a reduction > 70%				〇		〇		〇
Right								
Initial waveform	3608	1239	1423	1046	4253	343	59	114
Postoperative waveform	1370	1085	×	500	1085	355	131	×
a reduction > 70%					〇			

The MEP amplitudes during the initial and postoperative recordings are shown. “Initial” refers to baseline Tc-MEPecordings obtained after the induction of anesthesia and before the start of surgical manipulation. “final” refers to the final intraoperative Tc-MEP recordings obtained at the end of surgery, after completion of spinal correction and before emergence from anesthesia. All values are expressed in microvolts (µV). In postoperative recordings, the right quadriceps and anal sphincter muscles showed poor signal acquisition. Overall, the amplitudes of the right-side waveforms were larger. A > 70% reduction in amplitude was observed in the left hamstring, gluteus, sphincter, and right tibialis anterior muscles

*Deltoid* deltoid muscle, *ADM* abductor digiti minimi muscle, *Quad* quadriceps femoris muscle, *Ham* hamstring muscles, *TA* tibialis anterior muscle, *Gc* gastrocnemius muscle, *AH* abductor hallucis muscle, *Sphincter* external anal sphincter

The surgery was completed uneventfully. No changes were observed in the planned fixation range. Toward the end of the procedure, the doses of remimazolam and remifentanil were gradually tapered. Spontaneous movements were observed 29 min after remimazolam discontinuation; however, spontaneous respiration had not resumed. Flumazenil was administered in incremental doses (0.5 mg), after which spontaneous breathing resumed 9 min later, allowing for extubation. Immediately after extubation, upper airway obstruction due to tongue base collapse was noted, and a nasopharyngeal airway was inserted. The anesthesia time was 7 h and 26 min, and the surgical time was 4 h and 35 min. The total fluid balance was + 2083 mL. The estimated blood loss was 697 mL, urine output was 300 mL, and total fluid infusion volume was 2000 mL. The blood transfusion volume was 1080 mL, consisting of two units of packed red blood cells, 300 mL of salvaged autologous blood, and 500 mL of 5% albumin solution.

Postoperatively, the patient was admitted to the surgical intensive care unit (ICU). After admission to the ICU, opioids were avoided to maintain spontaneous respiration, and acetaminophen was administered as needed for analgesia. From the evening dose, 3 h after admission, the patient’s regular antiepileptic medications (clonazepam, perampanel hydrate, and levetiracetam) were resumed. That night, non-invasive positive-pressure ventilation, used regularly at home, was applied. His oxygen saturation transiently dropped to 85%, but improved with repositioning and suctioning. During this period, the respiratory rate was approximately 20 breaths/minute. The following day, his respiratory and circulatory statuses stabilized, and he was discharged from the ICU. No new paralysis was observed after surgery, and both the degree of spontaneous movement and the range of motion were preserved preoperatively. Postoperatively, the pain appeared to be adequately controlled with acetaminophen alone, without signs of severe distress or agitation.

## Discussion

The novelty of this case report lies in the anesthetic management of a patient with LS who underwent scoliosis surgery requiring MEP monitoring, in which remimazolam was selected as a balanced anesthetic option to address procedure- and patient-specific constraints in a metabolically vulnerable condition while preserving MEP signals.

In this case, several anesthetic considerations warrant attention. First, the metabolic vulnerability related to mitochondrial dysfunction, including susceptibility to lactic acidosis under anesthetic stress, must be considered. Since the 1985 report of malignant hyperthermia during general anesthesia in a pediatric patient with mitochondrial disease, anesthesiologists have generally avoided the use of volatile anesthetics [10]. However, recent reports indicate that there is insufficient evidence to support a definitive causal relationship between mitochondrial disease and malignant hyperthermia [5, 11], and no cases have been reported in patients with LS [12]. Furthermore, several reports have documented the safe use of inhalational anesthesia in patients with mitochondrial disease [13, 14]. Nevertheless, lactic acidosis has been reported to occur more frequently with volatile anesthetics than with propofol in patients with mitochondrial disorders [10], suggesting that their use requires careful consideration in metabolically vulnerable patients. In addition, some inhalational anesthetics exhibit epileptogenic properties that should also be taken into consideration [15].

Second, mitochondrial disease has been recognized as a risk factor for PRIS [16]. In fact, there have been reports of PRIS occurring in such patients [17]. Furthermore, prolonged propofol administration in pediatric patients in general has been reported to increase the risk of PRIS [18–21]. However, there are also reports describing the safe use of propofol in patients with mitochondrial diseases [13, 22–27]. Although such safe cases have been documented, and propofol remains a potential option, in surgeries expected to be prolonged, such as scoliosis correction, its use requires careful consideration, and alternative anesthetic strategies may be considered depending on patient-specific factors.

Third, taking these considerations together, anesthetic selection in the present case was guided primarily by patient- and procedure-specific factors rather than by the theoretical risks of individual agents. In this patient, long-segment posterior spinal fusion from T3 to the pelvis was planned for severe neuromuscular scoliosis. Reliable intraoperative motor-evoked potential (MEP) monitoring was considered essential because of the extent of correction and the associated risk of spinal cord injury. In addition, the patient had a markedly reduced respiratory reserve due to the thoracic deformity, making delayed emergence and prolonged postoperative ventilation particularly undesirable. Therefore, remimazolam was selected as a balanced alternative that allowed preservation of MEP signals [28–30] and offered flexibility in postoperative management through the availability of pharmacological reversal.

Other sedative agents such as ketamine, midazolam, and thiamylal are also available. Ketamine has been reported to have minimal suppressive effects on MEP amplitudes and has been successfully used for MEP monitoring during spinal surgery [31]. However, ketamine is characterized by a long effect-site concentration half-life and lacks a specific pharmacological antagonist, which may contribute to delayed emergence and prolonged postoperative sedation, particularly after long-duration surgery. In addition, ketamine may cause increased oral secretions and sympathetic stimulation, which could complicate postoperative airway and respiratory management in patients with a limited respiratory reserve. In the present case, which was characterized by central nervous system dysfunction and reduced respiratory reserve, the risks of delayed emergence and respiratory depression leading to prolonged intubation were of concern; thus, ketamine was not used. Midazolam, a benzodiazepine-like remimazolam, was also avoided because drug accumulation may increase the risk of delayed emergence during prolonged surgeries. Moreover, there is less evidence regarding its influence on MEPs compared to remimazolam, and it was therefore considered undesirable in this case. Thiamylal was also avoided because of concerns regarding drug accumulation. In contrast, remimazolam has a predictable pharmacokinetic profile and can be antagonized by flumazenil, allowing greater flexibility in postoperative emergence and airway management. Taken together, when balancing MEP signal preservation, postoperative respiratory safety, and controllability of anesthetic depth, remimazolam was considered the most suitable intravenous anesthetic option for this patient.

Based on these considerations, remimazolam was selected as the anesthetic in the present case. This case offers valuable insights into the anesthetic management of patients with mitochondrial encephalomyopathies, including LS, using remimazolam.

In the present case, intraoperative MEP monitoring was considered essential because the patient had severe neuromuscular scoliosis with a large Cobb angle and pelvic obliquity and underwent long-segment posterior fusion from T3 to the pelvis. Considering the extent of deformity correction and the associated risk of spinal cord injury, continuous neurophysiological monitoring was deemed necessary. Although alarm criteria for MEP monitoring vary, ranging from an irreversible amplitude reduction of 50–90% to complete waveform loss [32–36], our institution adopts a threshold of 70% reduction. In this case, asymmetry in the MEP amplitudes between the upper limbs, loss of signals from the right quadriceps femoris and anal sphincter, and amplitude reductions in both lower limbs were observed, meeting the predefined criteria (Table 1). No postoperative motor deficits were observed. The asymmetry in the upper limb amplitudes was likely influenced by a pre-existing contracture on the left side. The disappearance of signals from the right quadriceps and anal sphincter occurred approximately one hour after the start of the surgery, suggesting possible electrode displacement. Contracture of the right lower limb may have also contributed to difficulty in maintaining proper electrode positioning. The amplitude reduction may have been caused by hemodynamic changes, blood loss, or surgical manipulation. During surgery, blood transfusions and vasopressors were administered as needed to prevent hypotension and anemia. Given that no significant changes were observed in the upper limb muscles, which were less affected by surgical manipulation, the influence of remimazolam on the MEP amplitudes was likely minimal. Overall, this case suggests that remimazolam enables reliable intraoperative MEP monitoring while avoiding the potential risks associated with volatile anesthetics and propofol.

In the present case, the intraoperative SedLine values frequently remained in the 20s to low 30s, indiccating a deep level of anesthesia. However, the suppression ratio remained at 0% throughout the procedure, indicating that no burst suppression occurred. This finding supports the interpretation that excessively deep anesthesia was unlikely during surgery. However, considering the patient’s severe neurological impairment associated with Leigh syndrome, these values should be interpreted with caution. It has been reported that patients with mitochondrial encephalomyopathies accompanied by central nervous system disorders exhibit abnormal electroencephalographic (EEG) activity, and there is no evidence supporting the validity of BIS values, which are calibrated based on normal brain activity, in such patients [37]. Although SedLine monitoring was used, its numerical values were not the sole determinant of anesthetic depth. Therefore, maintenance with a relatively high remimazolam dose may have contributed to delayed emergence, and the possibility of excessive sedation cannot be completely ruled out.

When using remimazolam in patients with LS, the primary concern is delayed recovery of consciousness and respiratory function after surgery, as well as the potential for re-sedation and seizure occurrence. Previous studies [38, 39] have demonstrated that awakening is slower with remimazolam alone than with remimazolam combined with flumazenil and propofol. In contrast, the recovery profiles in the remimazolam with flumazenil group were comparable to those of the propofol group. Therefore, in patients with LS, flumazenil may facilitate recovery similar to that achieved with propofol alone. However, flumazenil carries risks of serious adverse effects, including sedation and seizures [40–43]. In the present case, the patient was taking clonazepam, and flumazenil administration could have antagonized its effects, thereby increasing the risk of seizure onset. At that time, continued sedation with endotracheal intubation and transfer to the ICU were also considered as alternative management strategies. However, given the patient’s severely reduced respiratory reserve due to neuromuscular scoliosis and thoracic deformity, prolonged postoperative intubation was considered to carry a substantial risk for respiratory complications, including difficulty with subsequent extubation, progression to tracheostomy, and ventilator-associated pneumonia. Consequently, early extubation was deemed more beneficial, and flumazenil was administered to prioritize the recovery of consciousness and respiratory function.

Postoperatively, the patient was closely monitored in the ICU, and regular antiepileptic medications were resumed in the evening. No postoperative seizures occurred. However, a transient episode of oxygen desaturation was observed. As discussed above, the interpretation of EEG-derived indices, such as the SedLine, requires caution in patients with Leigh syndrome, and delayed emergence and re-sedation remain potential concerns when remimazolam is used.

As described above, when using remimazolam in patients with Leigh syndrome, there are potential risks of delayed recovery of consciousness and respiration, re-sedation, and seizure induction, which may have been avoided with propofol. Careful attention should be paid to these adverse effects when remimazolam is used, and strict postoperative management in the ICU is recommended.

In the present case, the use of remimazolam enabled successful MEP monitoring while mitigating the risks of malignant hyperthermia and PRIS. Remimazolam may be considered a feasible anesthetic option for spinal surgery in patients with Leigh syndrome. However, in patients with impaired consciousness or respiratory function, there is a risk of delayed emergence, re-sedation, and seizure induction. Therefore, its use should be carefully determined by considering factors such as the patient’s age, level of consciousness, respiratory condition, and history of epilepsy, while weighing these risks against the potential for PRIS associated with propofol.

## Data Availability

Not applicable.

## Abbreviations

ICU: Intensive care unit
LS: Leigh syndrome
MEP: Motor evoked potential
PRIS: Propofol infusion syndrome
